# Biosorption: An Interplay between Marine Algae and Potentially Toxic Elements—A Review

**DOI:** 10.3390/md16020065

**Published:** 2018-02-19

**Authors:** Muhammad Bilal, Tahir Rasheed, Juan Eduardo Sosa-Hernández, Ali Raza, Faran Nabeel, Hafiz M. N. Iqbal

**Affiliations:** 1 State Key Laboratory of Microbial Metabolism, School of Life Sciences and Biotechnology, Shanghai Jiao Tong University, Shanghai 200240, China; bilaluaf@hotmail.com; 2 State Key Laboratory of Metal Matrix Composites, The School of Chemistry & Chemical Engineering, Shanghai Jiao Tong University, Shanghai 200240, China; masil@sjtu.edu.cn (T.R.); frnnbl@sjtu.edu.cn (F.N.); 3 Tecnologico de Monterrey, School of Engineering and Sciences, Campus Monterrey, Ave. Eugenio Garza Sada 2501, Monterrey, N.L., CP 64849, Mexico; eduardo.sosa@itesm.mx; 4 School of Biomedical Engineering, Shanghai Jiao Tong University, Shanghai 200240, China; aliraza86@sjtu.edu.cn

**Keywords:** biosorption, biosorbent, marine algae, toxic elements, environmental pollution

## Abstract

In recent decades, environmental pollution has emerged as a core issue, around the globe, rendering it of fundamental concern to eco-toxicologists, environmental biologists, eco-chemists, pathologists, and researchers from other fields. The dissolution of polluting agents is a leading cause of environmental pollution of all key spheres including the hydrosphere, lithosphere, and biosphere, among others. The widespread occurrence of various pollutants including toxic heavy metals and other emerging hazardous contaminants is a serious concern. With increasing scientific knowledge, socioeconomic awareness, human health problems, and ecological apprehensions, people are more concerned about adverse health outcomes. Against this background, several removal methods have been proposed and implemented with the aim of addressing environmental pollution and sustainable and eco-friendly development. Among them, the biosorption of pollutants using naturally inspired sources, e.g., marine algae, has considerable advantages. In the past few years, marine algae have been extensively studied due to their natural origin, overall cost-effective ratio, and effectiveness against a broader pollutant range; thus, they are considered a potential alternative to the conventional methods used for environmental decontamination. Herein, an effort has been made to highlight the importance of marine algae as naturally inspired biosorbents and their role in biosorption. Biosorption mechanisms and factors affecting biosorption activities are also discussed in this review. The utilization of marine algae as a biosorbent for the removal of numerous potentially toxic elements has also been reviewed.

## 1. Introduction

Increasing pollution is one of the major problems that our ecosystem is facing both at the aquatic and the terrestrial level. Some of the sources of polluting agents include chemical byproducts, herbicides, pesticides, pharmaceuticals, cosmeceuticals, leather, textiles, the plastic industry, pigments, electroplating, storage batteries, mining, smelting, metallurgical processes, nanoparticles, etc. [1,2]. However, besides their technological importance, the abovementioned contaminants are included in the category of persistent and/or emerging environmental pollutants, because they cannot be destroyed or degraded [1,3,4]. Also, these polluting agents are a leading cause of environmental pollution in almost all the key spheres, including the hydrosphere, lithosphere, and biosphere [2,5]. In recent decades, environmental pollution has emerged as a core issue around the globe, rendering it of fundamental concern to eco-toxicologists, environmental biologists, eco-chemists, pathologists, and researchers from other fields. Owing to rapid industrial expansion and the continued growth of the human population, the consequences of environmental pollution are worse than ever. Therefore, advanced methods with unique redefining approaches are required to meet the demands of the growing world population. Such strategies of the so-called “green agenda” are nowadays equally important for the sustainable development of all modern societies.

So far, numerous detection and removal methods have been proposed and implemented with the aim of addressing environmental pollution and sustainable and eco-friendly development. Among them, fluorescent-based sensors and electrochemical sensors for detection purposes, immobilized enzyme-based catalytic systems and photocatalytic systems for degradation purposes, along with other physiochemical-based process, etc. are the most widely used, though each have certain advantages and disadvantages [1,4,5,6,7,8,9,10,11,12,13,14,15,16,17,18,19,20]. Therefore, advanced methods with unique redefining approaches are required to remove potentially toxic elements and other hazardous pollutants from aqueous environments. Considering the adverse health outcomes, a clean and sustainable environment is of great importance.

The recovery of potentially toxic elements such as silver, gold, and uranium from chemically treated aqueous solutions is cost-effective [21]. However, the treatment of potentially toxic elements in wastewater is a challenge not only from an economic point of view but also from an environmental one. In order to remove potentially toxic elements, various physiochemical techniques such as ion exchange [22], chemical precipitation [23], electrokinetics [24], adsorption [25], and membrane processing [26] have been employed. Physiochemical processes pose high costs due to the expensive chemicals required for treatment of potentially toxic elements. Incomplete removal of potentially toxic elements is another factor involved in physiochemical processes. Furthermore, stringent conditions placed by regulating authorities on effluents also demand alternative methods. Potentially toxic elements can be removed from wastewater through biosorption from algae, which is an efficient, safe, and more economical method. Algae are also used for sorption of heavy radionuclides [27] and can recover metal ions such as gold and silver [28]. However, removal of potentially toxic elements can be attained by acquiring knowledge of algae. Biosorption mechanisms for potentially toxic element removal include ion exchange and complex formation, whereas electrostatic interaction proceeds at the micro-level. For the biosorption of metals, ion exchange is an important mechanism [29,30].

**Figure 1:**
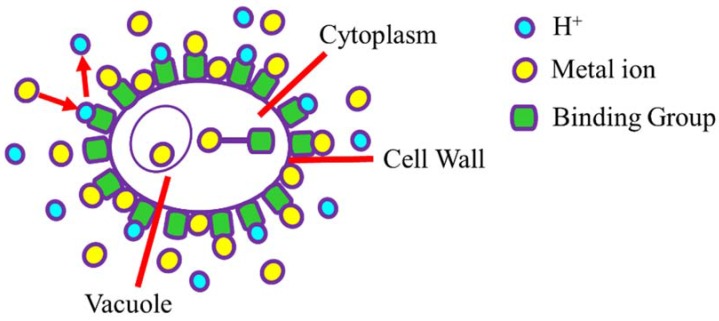
Biosorption of potentially toxic elements by an algae cell. Reproduced with modification from [38], with permission from Elsevier.

In this context, naturally inspired sources/materials, e.g., the biosorbent potentialities of marine algae and algae-based compounds, have appeared as an alternative technology with special reference to biosorption (Figure 1). Natural sources are now considered ecologically safer, cheaper, and more efficient for removing toxic metal ions and other hazardous pollutants from aqueous environments, e.g., industrial wastewater [15]. As compared to other methods, biosorption is rapid, reversible, economical, and eco-friendly. Owing to its range of novel aspects, as mentioned above, biosorption can be used in several ways under numerous conditions to reduce environmental pollution. Naturally inspired biosorbents, e.g. marine algae, offer several advantages such as (1) diverse multifunctional groups on their surface, (2) relatively small and uniform distribution of binding sites on the surface, (3) requires minimal preparatory steps, (4) no or less consumption of harsh chemicals, (5) naturally renewable, recyclable, and easily available all year round, (6) excellent retention capacity, and so on. Based on the literature, the utilization of marine algae as biosorbents has been successfully tested for several biotechnological and industrial applications including the removal of various potentially toxic elements [15,31,32,33,34,35,36,37].

## 2. Marine Algae: Sources, Production Strategies, and Applied Perspectives

Marine life is full of biodiversity but little explained in terms of total species. Indeed, there exists a range of exceptional sources of microorganisms including plants and animals that show particular features. Algae are one such group and cover about 30,000 species from various phylogenetic groups. Broadly, algae can be categorized into two groups: (1) macroalgae (multicellular); and (2) microalgae (unicellular). However, algae are heterogeneous from an evolutionary point of view [39]. Coastal area algae are mostly macroalgae and classified into three types: Chlorophyred (green), Rhodophyceae (red), and Phaeophycea (brown) algae [34,35,37,40,41]. Microalgae are found as phytoplankton throughout the oceanic ecosystem [40]. Microalgae consist of varied species and present biochemical properties, particularly undertaking oxygen-mediated photosynthesis [42]. They can survive under less favorable conditions such as high salinity, heat, cold, varied light sources, osmotic pressure, and anaerobiosis. Microalgae are the main food producers for animals because of fatty acid, sterol, carbohydrate, mineral, vitamin, protein, tocopherols antioxidant, chlorophyll, and carotenoid production [43].

During photosynthesis, algae cells convert solar energy to chemical energy, which generates chemical compounds of biological activities known as bioactive compounds. Production of these bioactive compounds could be associated with microbial and algal growth [44]. The *Dictionary of Marine Natural Products* is a key data source, listing more than 30,000 bioactive compounds from algae, and that number is gradually increasing every year [45].

Production of photosynthetic microorganisms by autotrophic cultivation is increasing, mainly for to get useful biomolecules. Various methods have been developed for microalgae cultivation such as open pond cultivation and photobioreactor cultivation, including membrane, flat-plate, helicoidal, horizontal, and vertical photobioreactors [46]. For improved biomass composition, tubular reactors using airlift systems are most common. Heterotrophic conditions could also produce microalgae with sufficient nutrients, whereas in mesotrophic environments and the non-availability of light, nutrients are achieved heterotrophically as well as autotrophically [47].

Macroalgae, also known as seaweeds, are used for the production of phycocolloids such as agar and alginates [48]. Some macroalgae like brown and red algae are employed in cosmetics because of their sugar, mineral, lipid, vitamin, and amino acid contents, as well as some other biological compounds [48]. Algae have become a sustainable resource due to the growing demand for bioprocessing to obtain environmentally friendly products [49]. Recently, bioactive compounds from algae [37,48], have become important and are being utilized in various environment-related applications like biofuel production, CO_2_ sequestration, wastewater treatment, oxygen discharge to the environment, and reducing the effects of greenhouse gases [40,50].

## 3. Biosorption and Its Mechanisms

**Figure 2:**
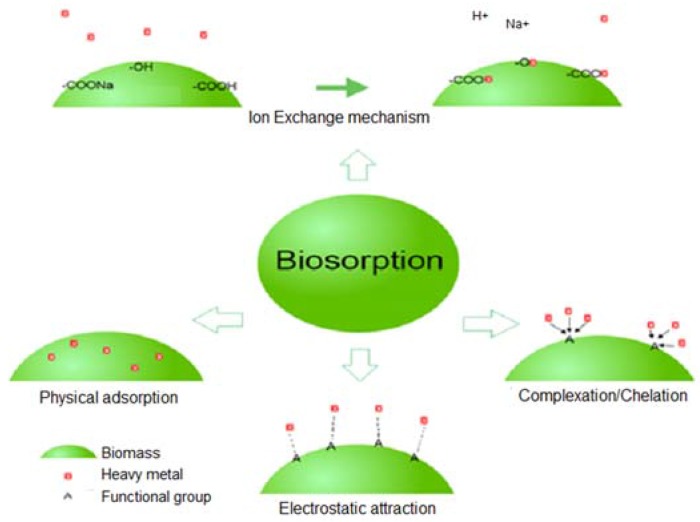
A schematic representation of the mechanisms involved in the biosorption of potentially toxic elements, e.g., heavy metal ions.

Different mechanisms have been reported for the removal of potentially toxic elements through biosorption. Previously, biosorption was categorized into metabolism-dependent or metabolism-independent biosorption [51]. However, later on, metabolism-dependent processes were termed bioaccumulation, and metabolic-independent processes were termed biosorption [52,53]. Bioaccumulation, also termed active biosorption, involves two processes: the first is similar to biosorption, involving attachment of potentially toxic elements to the surface; and in the second step active transportation of metal ions into cells occurs [54]. Biosorption is a passive process that occurs at a faster rate than bioaccumulation. Adsorption, chelation/complexation, ion exchange, and surface precipitation are different processes reportedly involved in biosorption (Figure 2) [55,56]. Among them, ion exchange is considered the principle mechanism of biosorption, which occurred through different functional groups present on the surface of biomass [53,57]. The mechanism of biosorption usually depends upon the biomass that is going to be used for the removal of potentially toxic elements [53]. For instance, the composition of the cell wall is different in bacteria (peptidoglycan), fungi (chitin), and algae (alginate, sulfonated polysaccharides); therefore, variation in the presence of functional groups on the surface of the cell wall is responsible for the difference in mechanisms [55,58]. Apart from the cell wall, extracellular polymer substances secreted by microorganisms are also found to play an important role in biosorption [59]. Verma et al. [60] reported ion exchange as a principle mechanism for the biosorption of potentially toxic elements by dry biomass of macrophytes. They presumed that aquatic macrophytes served as natural ion exchangers. Monovalent ions (H^+^, Na^+^/K^+^) were reported to be involved in the ion exchange process due to a weak attachment with biomass as compared to divalent ions [60]. Very recently, Ahmad et al. [61] reported that sulfate, carboxyl, and hydroxyl groups are involved in biosorption by microalgae. Changing of the morphological structure observed by scanning electron microscope (SEM) and/or energy dispersive X-ray (EDX) after biosorption also indicated the facilitation of sorption of metal ions by pores present on the surface [61]. However, in general, the mechanism of metal ion absorption by biomass is complicated and involves different processes.

## 4. Factors Affecting Biosorption 

Different factors can influence biosorption processes such as pH, temperature, initial metallic concentration, contact time, competing ions/co-ions, and biosorbent dosage [38,61,62]. Among these factors, pH is the most important factor. Increase in pH also increases the biosorption of metal ions, however; too great an increase in pH can cause precipitation, which should be avoided [62]. Optimum pH varied for different biosorption systems. An increase in pH up to 5 caused an increase in biosorption capacity (98%), but a further increase in pH led to reduced capacity. Protonation and deprotonation of functional groups is controlled by the pH of the medium, which affects the biosorption capacity: at low pH, carboxylic groups, being acidic, exist in a protonated state due to the presence of excess H^+^ and H_3_O^+^; therefore, repulsive forces of these protonated groups with positively charged heavy metal ions are responsible for the lower biosorption capacity at low pH [61,63]. With the increase in pH, functional groups such as amine, carboxyl, and hydroxyl groups are exposed by deprotonation, which enhances electrostatic attraction with heavy metal ions due to a negative charge. The high increase in pH leads to the formation of hydroxide anionic complexes and precipitation is a reported reason for the low biosorption capacity [61,63,64,65].

Temperature is also an important parameter influencing the sorption process [55]. Change in temperature alters thermodynamic parameters, resulting in variation in sorption capacity [38]. The influence of temperature on the sorption process depends upon its nature. In endothermic sorption processes, an increase in temperature leads to an increase in biosorption; on the other hand, an increase in temperature decreases biosorption in the case of exothermic sorption processes [38]. For instance, the biosorption of Pb(II) by algae was found to increase with an increase in temperature [66]. The biosorption of Pb(II), Cd(II), and Co(II), from an aqueous solution on green algae waste biomass has also been reported [16]. In another report, Ahmad et al. [61] found a reduction in biosorption of Fe(II), Mn(II), and Zn(II) by freely suspended and Ca–alginate immobilized with *Chlorella vulgaris* with an increase in temperature (25–45 °C) due to the exothermic nature of the biosorption process. A very high temperature can also denature the biomass structure [61].

The contact time of biosorbent influences total biosorption. An increase in contact time up to the optimum contact time increases biosorption; afterwards it becomes relatively constant. Occupancy of all active sites causes saturation of biomass, leading to an equilibrium state [63,67]. The optimum time is different for different types of biosorbents such as 60 min for red macroalgae [63] and 300 min for immobilized algal mass (240 min for free suspended mass) [61]. The initial metal ionic strength also affects biosorption. A high initial metallic concentration exhibits high biosorption capacity due to the availability of free active sites [68]. A similar trend was also reported by changing the adsorbent dose, i.e., initial increase and then equilibrium state [69,70].

## 5. Potential Biosorbents

For biosorption purposes, different living and non-living biomasses have been reported such as algal biomass, fungi, bacteria, agricultural waste, etc. [71]. Typically, an ideal biosorbent should possess features like availability, non-toxicity, high metal binding capacity, large-scale usability, and regeneration/re-usability [21]. Algal biomass is the most employed biosorbent compared to any other material. As no treatment is required for algae, it is considered a low-cost biosorbent, and its cell wall characteristics endow it with high metal ion binding capacity [72]. Non-living algal mass has been reported to be more promising as compared to living algae because of the higher metal ions sorption capacity at a higher rate, and it does not require nutrients grown in a medium [38]. Moreover, adsorbed heavy metal ions on dead algal mass can be removed using de-ionized water or desorption agents [38]. Different types of algae have been reported as biosorbents such as marine algae, marine red macroalgae, marine brown macroalgae, and freshwater green macroalgae. Brown algae are reported to have good biosorption capacity due to the presence of alginates in their cell walls [73].

## 6. Potentially Toxic Elements—Heavy Metals

Heavy metals exist naturally in the earth’s crust and are distributed in the soil. While there is no clear definition for heavy metals, density is the defining element in most situations. Conventionally, on the periodic table, heavy metals are those with an atomic number greater than 20 [65]. Considering their atomic density higher than 4 g cm^−3^ (5 times higher than water), they form a group of about 53, including metals and elements with metallic properties [74]. Heavy metals are referred to as high-density elements and cause toxicity at low strengths. Nevertheless, from an ecological point of view, any metal or related metalloid that is not biodegradable and produces environmental pollution can be taken as a heavy metal [74]. Some heavy metals (e.g., Cu, Zn, Ni, Cu, Mn, and Co) are essential for plant life, whereas some do not have any biological role and have toxic effects (e.g., Pb, Hg, and Cd) [75]. Therefore, any metal or metalloid that causes a harmful effect on the environment, does not play a vital role in biological functions, shows toxicity at low strengths (e.g., Pb and Hg), or plays a vital role in biological functions but produces harmful effects if present in a high concentration (e.g., Cu and Mo) [74]. Wang and Chen [21] classified harmful heavy metals into three divisions: toxic metals (Pb, Hg, Zn, Cr, Ni, Co Cd, Cu, Sn, As, etc.), radionuclides (U, Th, Am, Ra, etc.), and precious metals (Au, Ag, Pt, Ru, etc.).

Urbanization, industrialization, population growth, and continuous cultivation have damaged the global environment. The toxic effects and major mechanisms of toxicity of different heavy metals are summarized in Table 1. Human activities and industrial applications of heavy metals have increased toxicity levels and pose harmful effects to the environment [74,76]. Several reports report the varied use of heavy metals [77] in different industrial [21] and agrarian practices [78], in addition to indiscriminate disposal [79]. Among the other harmful effects of heavy metals, water pollution is one of the serious issues worldwide [80]. Several heavy metals released into the environment by different activities undergo transformations, are dispersed and accumulated in the food chain, and cause serious health concerns [81]. In short, removal of heavy metals is a challenge as they are not biodegradable and hence hinder the self-purification capability of marine life when discharged to the aquatic environment [82].

## 7. Biosorption of Potentially Toxic Elements

Potentially toxic elements are removed from wastewater by algae, mostly through inactive biomass and non-living algae. Fewer data are available on using live algae for potentially toxic element removal [103], as toxic elements poison the algae. Although sorption largely depends on the stage of the algae growth, there are several factors that affect the biosorption of metal ions. The process of biosorption is more complicated in living algae as compared to non-living algae as biosorption occurs in the growth stage and the intracellular consumption of potentially toxic elements mostly takes place at this stage. The cells of non-living algae absorb potentially toxic elements at the cell membrane surface. Therefore, the process is known as an extracellular process [104]. Non-living algal biomass includes polymers such as sugars, glycoproteins, cellulose, pectins, etc., which effectively bind and adsorb potentially toxic elements from wastewater [105]. Two stages are involved in the accumulation of potentially toxic elements through microorganisms [106]. During the first stage, fast inactive biosorption proceeds at the cell surface with no cellular metabolism involved, whereas active sorption occurs during the second stage, which involves the cell cytoplasm. The second stage is considered intracellular ion accumulation since it involves cell metabolism. Intercellular ion accumulation plays a vital role in the biosorption detoxification of potentially toxic elements [107].

**Figure 3:**
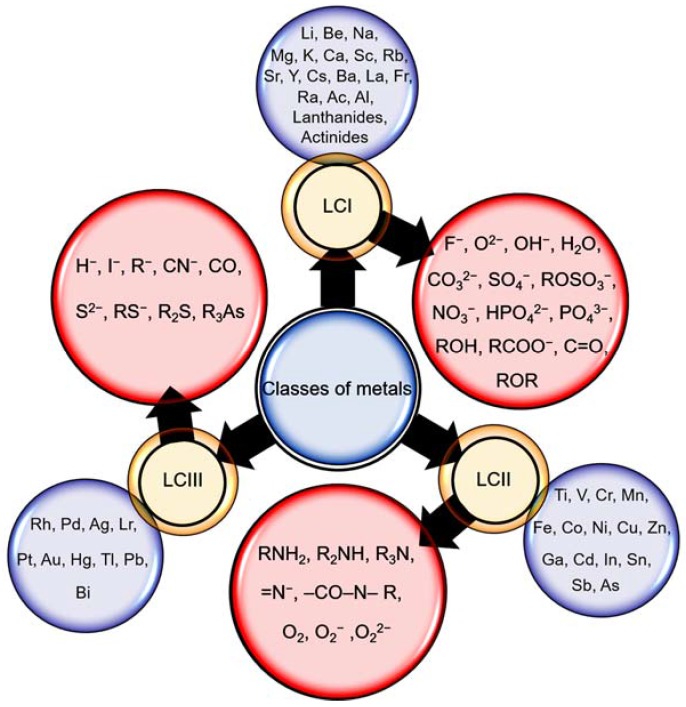
A schematic representation of three classes of metals based on ligands present in biological systems. LCI, ligand class I; LCII, ligand class II; and LCIII, ligand class III.

The biosorption ability of algae cell surface is designated by the availability of the binding moiety, i.e., carboxyl, hydroxyl, amine, phosphoryl, sulphuryl, sulfate, carbohydrate, imidazole, phosphate, etc. [108]. The presence of the binding site on the algae cell for potentially toxic elements accumulation is analyzed by Fourier-transform infrared spectroscopy (FTIR) spectroscopy [109]. Accumulation of the potentially toxic elements on the algae cell depends on various factors such as the number of functional moieties, the approachability of binding sites, binding constants, and the chemical state of the moieties. Most binding moieties make the cell surface negatively charged overall as a result of carboxyl and phosphate deprotonation [110]. Biosorption of the metal ions starts from the algae cell wall. Various binding groups, for example OH^−^, COO^−^, NO^3−^, RS^−^, SH^−^, PO_4_^3−^, RNH^2−^ and RO^−^ encourage adsorption of the metal ions. These groups exist outside (cell surface) and inside (cytoplasm and vacuoles) the cell wall. The biosorption mechanism of metal ions across the cell walls could be supported by cytosolic protein-mediated metal ions transfer [111]. Vacuoles, therefore, accumulate metal ions and are considered metal ion organelles. The data based on the affinity for various metal ions and the corresponding cellular ligands are illustrated in Figure 3. Ligands with R present alkyl groups, for example propyl and metal ions categorized into A, B, and borderline subcategories. Category I shows the connection of ligands with metal ions of Class A through an oxygen atom. Metal ions from Class B can be connected with ligands of category II and III, whereas borderline cations can be associated with various atoms from categories I, II, and III [52]. The categorization is well defined, but it would be more convenient if the classification is based on metal complexation constants from a chemistry viewpoint. This can be more helpful for scientists trying to decide on the biosorption ability of a particular metal among competing ions.

The cell wall in algae cells is an initial hindrance to the biosorption of potentially toxic elements. Most of the binding sites present in algae cells are due to polysaccharides and proteins [112]. Different algal strains have different cell wall compositions and varying capacity of biosorption of potentially toxic elements. Romera et al. [113] studied different algae strains for the biosorption of potentially toxic elements and proposed brown algae as a good contender. Brown algae have a high affinity for the biosorption of lead because of the presence of alginate in the cell walls and the capacity of biosorption depends on the binding sites available on the alginate [32,113]. Several factors such as metal ion concentration, competing for metal ions, temperature, and pH affect the biosorption of potentially toxic elements in addition to available binding sites on the algae cell.

### 7.1. Biosorption of Cadmium

Cadmium is found in nature in the form of deposits with other elements. The toxic metal is discharged in industrial effluents from phosphate fertilizers, plating, stabilizers, and cadmium–nickel alloy batteries. Low concentrations of cadmium can accumulate and become harmful to the ecosystem. Cadmium can cause “Itai-itai” bone softening and fractures in humans [114]. Other effects of cadmium on human health include lung cancer, kidney failure, and damage to respiratory and reproductive systems [115,116]. Therefore, efficient, reliable, and economical removal of cadmium from water is required. Biosorption of cadmium using marine algae is a safe, useful, and eco-friendly solution to wastewater treatment. Table 2 presents various algae’s capacity to remove potentially toxic elements. The table comprises Cd^2+^ removal in the pH range from 4 to 8. It has been found that algae *C. reinhardtii*, *Scevedesmus* spp., *S. platensis*, *Chlorella* spp. as well as *Tetraselmis* spp. efficiently remove Cd^2+^, whereas the uptake of Cd^2+^ from *Plaothidium lanceolatum* live cells is significant, i.e. 275.51 mg g^−1^ [117].

### 7.2. Biosorption of Chromium

Chromium is the seventh most prevalent metal on earth and is found in ores with other metals like chromite (FeCr_2_O_3_), chrome ochre (Cr_2_O_3_), and crocoite (PbCrO_3_). The foremost industrial sources of chromium are the leather, tanning, textile, and electroplating industries. Industry waste has hexavalent and trivalent ions of chromium, i.e., Cr^6+^ and Cr^3+^ [129]. Cr^3+^ is less toxic to living organisms than Cr^6+^ [116,130]. Cr^3+^ is helpful in sugar and fat metabolism, and Cr^6+^ is used in industry for salt production [129,131]. Both states of chromium metals are used for chrome plating, pigment fabrication, in the glass industry, in the leather industry as a tanning agent, etc. [132]. They damage organs such as the liver, kidneys, and skin and can cause ulcers, pulmonary congestion, and vomiting [132,133]. In order to reduce the effects of chromium, it needs to be modified to a less toxic state and treated before release into the environment.

### 7.3. Biosorption of Lead

Lead is also a toxic metal that can easily accumulate in plants, the human body, and other living organisms. It is found in nature in the state of sulfide, cerussite (PbCl_2_) ore, and galena [134]. Wastewater from industries such as electrical, steel, electroplating, explosives, etc. is the main source of lead pollution. Its function is to produce DNA, protein, and replication of the cell [134]. It is harmful to the nervous system, kidneys, and mental health and causes cancer in humans [135,136]. Lead is also toxic to plants and animals. Therefore, the latest techniques are required to remove lead from water. Biosorption through algae is an innovative technique for the removal of lead from the ecosystem. As mentioned in Table 2, *Spirulina* as well as *Chlorella* ssp. algae show potential for Pb^2+^ removal capacity. Also, significant results have been achieved for lead removal with immobilized cells, i.e., *C. reinhardtii* [125].

### 7.4. Biosorption of Zinc

Zinc is helpful in various biochemical processes and controls physiological mechanisms in living tissues. With other metals (such as steel alloys), it works as a protective layer to control corrosion. Other forms of zinc are used in industrial processes like steel, mining, and coal combustion [137]. Although a trace amount of zinc is required by the body, if present in excess amounts it can upset normal health. It can cause fever, pain, skin inflammation, vomiting, and anemia [137]. Sources of zinc pollution in the ecosystem are the paper, steel, electroplating, and brass industries. Given the effects of zinc metals, it becomes important to treat wastewater effectively before releasing it into the environment. Zinc has been studied for biosorption in the pH range from 5 to 7.5 (Table 2). *P. lanceolatum* algae are a potential contender to remove zinc metal up to 118.66 mg g^−1^ [117].

## 8. Concluding Remarks and Future Considerations

In conclusion, the above data suggest that the effective removal of potentially toxic elements from aqueous media can be achieved by biosorption using marine algae biomass as a biosorbent. Naturally inspired biosorbents, e.g., marine algae, offer several advantages such as (1) diverse multifunctional groups on their surface, (2) relatively small and uniform distribution of binding sites on the surface, (3) requiring minimal preparatory steps, (4) no or less consumption of harsh chemicals, (5) being naturally renewable, recyclable, and easily available all year round, (6) excellent retention capacity, and so on.

Despite the modern expansion of the biotechnological and industrial arena, along with the plethora of information available on various types of bioremediation processes, numerous challenges still need to be addressed. For example, the emergence of new pollutants, potentially toxic elements’ dissemination profiles, eco-friendly detection, removal fate, and reliable and consistent monitoring are research gaps that need to be addressed in future studies. In this context, proper management and strategies should be adapted to maintain aquatic and terrestrial environmental health and protect from further deterioration.

Furthermore, many other unsolved questions must be tackled. For example, insufficient detection methods, malpractice, and limitations within practice technologies greatly affect the detection fate and removal behavior of potentially toxic elements. The role of low-risk contaminants in the emergence of new pollutants should also be addressed in future studies. It could be useful to involve multidisciplinary scientists, policymakers, and stakeholders to strengthen the detection and removal/degradation of life-threatening pollutants at a global level.

## Figures and Tables

**Table 1:** Toxic effects and major mechanisms of toxicity of different heavy metals.

Heavy Metal	Major Uses/Sources	Toxic Effects	Mechanism of Toxicity	References
Lead (Pb)	Lead batteries, lead paint, devices to shield from X-rays.	Nervous system, male reproductive system, microvascular endothelium, immune system, impairs mammalian spermatogenesis and sperm quality in vivo, inhibits sperm functions in vitro.	Lead has no biological functions. Oxidative stress (reactive oxygen species, ROS), with a reduction in the effects of antioxidants, is the principal mechanism. Lead ions also replace other ions such as Ca^2+^, Mg^2+^, and Na^+^ and disturb normal cell functions such as cellular adhesion, apoptosis, and neurotransmitter release.	[83,84,85,86,87,88]
Arsenic(Ar)	Agricultural chemicals (pesticides, fungicides, herbicides).	Cardiovascular/peripheral vascular disease, developmental abnormalities, immunological, and neurological disorders, carcinogenesis, diabetes, portal fibrosis.	Oxidative stress, genotoxicity, alteration in DNA repair, and p53 suppression (major contributor to carcinogenesis).	[89,90,91,92,93]
Cadmium (Cd)	Metal industry, paint pigments, fertilizers, cigarette smoke, food.	Pulmonary and gastrointestinal irritation, carcinogenesis (development of adenocarcinomas), Kidneys, liver and bones are also effected by cadmium exposure.	Competition with other ions (zinc, iron, copper), genotoxicity, lipid peroxidation, oxidative stress.	[94,95,96,97]
Chromium Cr(III)/Cr (VI)	Anticorrosive, industrial welding, chrome plating, leather industry, wood preservation.	Carcinogenic, gastric and intestinal ulcers, sperm damage, male reproductive system problems, anemia.	Cr (VI) is more potent than Cr (III); Oxidative stress, genotoxicity, alteration in cellular signaling pathway	[94,98]
Mercury (Hg)	Natural processes involved oceanic emissions and biomass burning. Anthropogenic sources included power plants, metal industry and gold mining.	Alzheimer’s disease, Parkinsonism, respiratory depression	Binding of mercury with sulfhydryl (–SH) groups disrupts normal cellular enzymatic processes. Increase in free radical concentration due to blockage of GSH by Hg is responsible for cell-damaging effects.	[94,95,97]
Copper (Cu)	Agriculture (fertilizers), leather industry (tanning), and photo-voltaic cells.	Carcinogenic, neurodegenerative disorders, responsible for complications in diabetes, promotes atherosclerosis.	Oxidative stress, enzyme inhibition, replaces normal ions of the body.	[94,99,100,101]
Zinc (Zn)	Oil refinery, mining, brass manufacturing, plumbing.	Ataxia, depression, gastrointestinal irritation, hematuria, icterus, impotence, kidney and liver failure, lethargy, macular degeneration, metal fume fever, prostate cancer, seizures, vomiting.		[94,102]

**Table 2:** Biosorption potentialities of various marine algae to remove potentially toxic elements from aqueous solutions.

Potentially Toxic Elements	Algae Used	Adsorption Capacity	References
Zn(II)	*Ulva* sp.	29.63 mg/g	[118]
Cd(II)	*Chlorella vulgaris* (dead)	96.8%	[119]
Cd(II)	*Chlorella vulgaris* (live)	95.2%	[119]
Cd	*Scenedesmus quadricauda*	66%	[120]
Pb	*Scenedesmus quadricauda*	82%	[120]
Cd(II)	*Ulva lactuca*	85%	[121]
Cd(II)	*Ulva lactuca*	29.2 mg/g	[122]
Pb(II)	*Ulva lactuca*	34.7 mg/g	[122]
Cd(II)	*Ceramium virgatum*	39.7 mg/g	[123]
Cu(II)	*Ulva fasciata*	73.5 mg/g	[124]
Cu(II)	*Sargassum* sp.	72.5 mg/g	[124]
Hg(II)	*Chlamydomonas reinhardtii*	89.5 mg/g	[125]
Cd(II)	*Chlamydomonas reinhardtii*	66.5 mg/g	[125]
Pb(II)	*Chlamydomonas reinhardtii*	253.6 mg/g	[125]
Cr(VI)	*Spirogyra* sp.	14.7 × 10^3^ mg metal/kg	[126]
Cd(II)	*Padina* sp.	90%	[127]
Cd(II)	*Durvillaea potatorum*	90%	[128]
